# Usage patterns and adverse experiences in traditional Korean medicine: results of a survey in South Korea

**DOI:** 10.1186/1472-6882-13-340

**Published:** 2013-12-01

**Authors:** Hyeun-Kyoo Shin, Soo-Jin Jeong, Dae Sun Huang, Byoung-Kab Kang, Myeong Soo Lee

**Affiliations:** 1 Herbal Medicine Research Division, Korea Institute of Oriental Medicine, 483 Expo-ro, Yusung-gu, Daejeon 305-811, South of Korea; 2 Medical Research Division, Korea Institute of Oriental Medicine, 483 Expo-ro, Yusung-gu, Daejeon 305-811, South of Korea

## Abstract

**Background:**

Although traditional medicine (TM) in South Korea is included in the national health care system, it is considered complementary and alternative medicine (CAM), and not mainstream medicine. Therefore, the lack of statistical data regarding the usage and adverse experiences of traditional Korean medicine (TKM) makes difficult to understand the current status of TM. In this study, we aimed to report usage patterns and adverse experiences on TKM targeting consumers in South Korea.

**Methods:**

A total of 2000 consumers participated in the survey on usage and adverse experiences in 2008. Among the 2,000 participants, 915 (45.8%) had taken herbal medicine or received traditional medicinal therapies; these individuals were further surveyed on the internet or in an interview.

**Results:**

The usage rate was higher among women and among patients in their 30s. Of the total TKM usage, acupuncture accounted for 36.7%, and herbal medicine accounted for 13.4%. Regarding the frequency of use of TKM, 73.8% of patients reported using TM less than 5 times in 1 year. Of the 915 respondents, 8.2% of individuals had some type of adverse experience resulting from TKM. Adverse experiences were primarily caused by acupuncture and herbal medicines, and they primarily involved diseases of the digestive system and skin. The incidence of adverse experiences was less than 3.7% for acupuncture and 3.8% for herbal medicine. Overall, the incidence rate of adverse experiences for TKM for the entire population was 0.04 per 10,000 individuals.

**Conclusions:**

The medical usage and occurrence of adverse events on TKM should be surveyed periodically, and the statistical trends should be analysed. The disparity between the survey results for traditional herbal medicines and medical practices, and those for the national pharmacovigilance system or academic reports of adverse experiences should be examined. The national pharmacovigilance system must be improved to compensate for the disparities. Policies and regulations are required to enhance the reporting of adverse experiences not only for herbal medicines but also for traditional medicinal therapies.

## Background

Recently, interest in complementary and alternative medicine (CAM) and traditional medicine (TM) has increased in the clinical settings of many countries, and herbal medicines and acupuncture have become globally popular therapies [1,2]. Herbal medicines have been used in the clinics of China, Korea and Japan for thousands of years, and the toxicity and efficacy of herbal therapies have been well established in the literature. These studies have become important sources of information and techniques for treating intractable diseases [3]. However, the largest problems regarding CAM/TM are the lack of a sufficient scientific basis for establishing efficacy and the questions that have arisen regarding the safety of increasing the use of CAM/TM.

Traditional Korean medicine (TKM), which is one part of CAM, faces similar problems. In 2008, 14,818 TKM doctors in South Korea treated a total of 12,128,657 patients with TKM that were reimbursable by the national health insurance system. These therapies included 56 types of herbal formulations and 68 types of single herbal crude extracts as well as traditional therapies, such as acupuncture, moxibustion, cupping therapy [4]. Since TKM was first included in the national insurance system, its usage rate has increased, whereas the number of reports of adverse events remains low.

The first case involving the adverse events of herbal medicine was reported at a conference in South Korea in 1979 [5]. The reporting of adverse events caused by all drugs and treatments for human diseases is an important topic for which the government has set specific regulations. The Korean government first established regulations of pharmacovigilance in the national public healthcare system in 1988, which required that all adverse drug events be reported according to the regulations [6]. In 2007 and 2008, the number of reported adverse events in South Korea was 3,750 and 7,210, respectively. Among these cases, only 8 cases were reported on herbal medicines in both 2007 and 2008 [7,8]. Adverse events of chiropractic therapy and acupuncture were first reported in 1988 and 2006, respectively [5,6]. Since then, reports of adverse events of TM therapy have been rare.

The current pharmacovigilance system does include a method for the spontaneous reporting of adverse events of herbal medicines; however, there are no systems in place for monitoring and reporting adverse events of other traditional therapy methods. Any adverse event that occurs can only be determined from publications reported by relevant experts. Thus, there are currently no methods for assessing the number of adverse events arising from herbal medicines or other traditional therapies in South Korea.

To recognise the increasing interest in traditional therapies among the public, this survey aims to assess the usage patterns and adverse experiences of TKM affecting consumers in South Korea.

## Methods

### Study design

This study is a survey of Koreans on usage patterns and adverse experiences in Korean medicine. We collected the participants for the survey by sex, age, and residence and designed a statistically number of samples.

### Setting

A total of 2,000 South Koreans (1,000 each, male and female) over the age of 20 were surveyed at the NI-Korea company between December 1 and December 17, 2008. We promoted the study to the public on the internet and interviewed individuals with trained interviewers from a professional survey research company. We randomly advertised the survey and sent a questionnaire by email to the study participants.

### Participants

We clearly determine the number of survey population (n = 2,000) using sample size calculation and stratified them by sex, age and residence. Then, we randomly sampled from the sampling frame provided by the Statistics Korea based on Neyman’s allocation method. Among the 2,000 individuals by sample size, 1,771 were surveyed online, while 229 individuals were interviewed face-to-face. If there is survey nonresponse, we randomly chose and added new sample from the sampling frame.

### Variables

The survey questions used are as follows:

– Experience and number of TKM usage events

– Usage patterns of TKM treatment (e.g., acupuncture, moxibustion, cupping therapy, physical therapy, and chiropractic) and herbal medicine

– Number of adverse experiences and the targeted organ of TKM

### Data sources/measurement

The symptoms of adverse experience were classified into five groups as follows: diseases of the digestive system, diseases of the skin, diseases of the nervous system, disorders of the kidneys, and other disorders.

### Bias

This study was a retrospective survey. Recall bias and selection bias may have existed in this study.

### Study size

A weighted application three-society stratified sample (sex, age, and area of administration) method was used for analysis by proportionate probability sampling. The stratified random sampling targeted a sampling error of ±2.2 with a confidence interval of 95%.

The total sampling number:

$$n=\frac{{\left(\sum \mathrm{N\sigma}\right)}^{2}}{{\left(\frac{\epsilon }{t_{\alpha }}\times \sum X\right)}^{2}+\sum N\sigma^{2}}$$

Sampling number in each class:

$$n_{s}=n\times \frac{\mathrm{N\sigma}}{\sum \mathrm{N\sigma}}$$

*n*: national sample population over the age of 20

*n*_*s*_: sample population in each class

*N*: national parent population over the age of 20

*X*: population in each class

*σ*: standard deviation of population in each class

*σ*^*2*^: variance of population in each class

$$\frac{\epsilon }{t_{\alpha }}$$

: expecting precision (ϵ = tolerance error, *t*_*α*_ = confidence coefficient).

The total of 2,000 (1,000 each, male and female) people over the age of 20 was calculated using Neyman’s sample number decision formula and the optimal allocation method. The 1,085 individuals who did not have any experience with TKM were excluded. The remaining 915 individuals who had experience with TKM were surveyed for the development of any adverse experience of TKM therapies. Thus, the survey was analyzed for 915 respondents.

### Statistical analysis

Frequency analysis was conducted for the questions. The survey results analyses were performed using SPSS WIN 12.0K (SPSS Inc., Chicago, IL, USA).

### Ethical considerations

The ethical review committee of the Korea Institute of Oriental Medicine waived the need for formal ethical approval and informed consent to be obtained from participants due to the nature of the study. The survey was already conducted on a voluntary basis with agreement from the participants for the use of the collected data for scientific purposes.

## Results

### Usage patterns of traditional Korean medicine

Of the initial 2,000 individuals surveyed, only 45.8% had received TKM, including herbal medicines, acupuncture, moxibustion, cupping therapy, physical therapy, or chiropractic therapy, during the previous year. The rates of usage for each TKM in proportion to the total 2000 individuals surveyed were as follows: acupuncture, 36.7%; herbal medicine, 13.4%; physical therapy, 10.0%; moxibustion, 9.0%; cupping therapy, 5.7%; chiropractic therapy, 1.5%; and other therapies, 0.5%.

Of the 915 individuals who had received TKM, more females (53.6%) used these TKMs than males (46.4%). Among the different age groups, TKM usage varied as follows: 26.7% of those in their 30s used TKM, 25.0% of individuals in their 40s used TKM, 21.2% of individuals in their 20s used TKM, 17.3% of individuals in their 50s used TKM and 9.8% of individuals in their 60s used TKM. In terms of the frequency of usage, 73.8% used TKM fewer than 5 times per year, 13.1% used TKM 6 to 10 times per year and 13.1% used TKM more than 11 times per year.

Moreover, 915 people who used TKM received combination therapies. Acupuncture was the most commonly repeated therapy at 80.1%, followed by herbal medicines (29.2%), physical therapy (21.9%), moxibustion (19.6%), cupping therapy (12.4%), chiropractic therapy (3.3%) and other therapies (1.1%).

Of the 425 males, 80.2% received acupuncture, 24.5% took herbal medicines, 21.6% received physical therapy, 19.2% received moxibustion, 13.8% received cupping therapy and 3.9% received chiropractic therapy. Of the 490 females, 80.1% received acupuncture, 33.2% took herbal medicines, 22.2% received physical therapy, 19.9% received moxibustion, 11.1% received cupping therapy and 2.7% received chiropractic therapy. The gender distribution for the use of each therapy was as follows: females comprised 53.5% of the total acupuncture users, 61.0% of the herbal medicine users, 54.0% of the physical therapy patients and 54.2% of the moxibustion users, all of which represented higher usage rates compared with the males. However, males represented 52.2% of the cupping therapy users and 56.7% of the chiropractic therapy users compared with women.

Regarding the age group distribution, 78.1% of the 194 individuals in their 20s received acupuncture and 30.9% took herbal medicines. Regarding the 244 individuals in their 30s who were surveyed, 77.2% received acupuncture and 31.8% took herbal medicines. Of the 90 seniors in their 60s, 90.0% received acupuncture and 45.3% took herbal medicines; both rates were higher than those of any other age group.

Regarding the type of therapy, of the 733 total acupuncture cases, the predominant recipients were people in their 30s (25.7%) and 40s (24.8%). Of the 267 individuals who used herbal medicine, 29.2% were people in their 30s. Regarding the 200 cases of physical therapy, 28.0% represented people in their 30s. Regarding the 179 cases of moxibustion, 26.8% were people in their 40s. Regarding the 113 cases of cupping therapy, 35.4% were individuals in their 30s. Regarding the 30 cases of chiropractic therapy, 30.0% were people in their 40s, who were the most frequent recipients of this type of therapy.

Of the total cases for each therapy, the percentage of individuals receiving 5 or fewer treatments of TKM were 71.6% for acupuncture, 73.8% for herbal medicines, 75.5% for physical therapy, 68.7% for moxibustion, 68.1% for cupping therapy and 70.0% for chiropractic therapy (Table 1).

**Table 1:** Usage of traditional Korean medicine (n = 915)

**Demographic characteristics**	**No. of respondents**	**Types of traditional Korean medicine**						
		**Acupuncture**	**Herbal medicine**	**Physical therapy**	**Moxibustion**	**Cupping**	**Chiropractic**	**Others**
All respondents	915	733(80.1)	267(29.2)	200(21.9)	179(19.6)	113(12.4)	30(3.3)	10(1.1)
Gender								
Male	425	341(80.2)	104(24.5)	92(21.6)	82(19.2)	59(13.8)	17(3.9)	3(0.7)
Female	490	392(80.1)	163(33.2)	108(22.2)	97(19.9)	54(11.1)	13(2.7)	7(1.4)
Age								
20-29	194	152(78.1)	60(30.9)	46(23.6)	41(20.6)	14(7.0)	4(2.2)	1(0.4)
30-39	244	188(77.2)	78(31.8)	56(22.9)	37(15.2)	40(16.6)	7(2.8)	3(1.2)
40-49	229	182(79.6)	53(23.3)	40(17.7)	48(21.1)	36(15.7)	9(4.0)	5(2.0)
50-59	158	130(82.3)	35(22.4)	34(22.2)	39(24.9)	10(6.5)	8(4.9)	1(1.0)
60-69	90	81(90.0)	41(45.3)	24(26.2)	14(16.0)	13(14.2)	2(2.3)	-
N. of utilizations								
≤5	675	525(77.9)	197(29.2)	151(22.5)	123(18.3)	77(11.4)	21(3.2)	7(1.0)
6-10	120	104(86.4)	35(29.4)	29(24.4)	27(22.2)	17(14.3)	3(2.1)	-
≥11	120	104(86.4)	35(29.0)	20(16.6)	29(24.1)	19(15.8)	6(4.6)	3(2.9)

All data are in n (%).

### Adverse experience from traditional medicinal therapies and herbal medicines

The 915 individuals included in this study received a total of 1,532 TKM treatments. When asked about adverse experiences, 7.2% of the individuals reported adverse experiences, while 91.8% individuals reported no adverse experiences. The types of adverse experiences included diseases of the digestive system (40.0%), skin (30.6%), nervous system (14.6%), kidney (6.6%) and others (7.9%).

Of the 425 male and 490 female participants, 8.7% and 7.9%, respectively, reported adverse experiences. In terms of the type of adverse experiences, diseases of the digestive system (43.2%) and skin (37.8%) were predominant in males. For the females, diseases of the digestive system (36.8%), skin (23.7%) and nervous system (23.7%) were the most common.

Respondents in their 20s had the highest rate of adverse experiences at 10.3%, while sexagenarians had the lowest rate of the experiences at 4.4%. Age distribution regarding adverse experiences on TKM was as follows: 20s (26.7%), 30s (28.8%), 40s (20.0%), 50s (20.0%) and 60s (5.3%). The most common type of adverse experience for each group was as follows: diseases of the digestive system for individuals in their 20s (60.0%), diseases of the skin for both individuals in their 30s (38.0%) and those in their 40s (40.1%), diseases of the digestive system for individuals in their 50s (60.2%) and diseases of the skin for individuals in their 60s (76.0%).

The increased use of TM therapy also led to an increase in adverse experiences, as the percentage was 7.7% in those receiving 5 or fewer treatments, 8.3% in those receiving 6 to 10 treatments and 10.8% in those receiving 11 or more treatments. The most common type of adverse experience for each group was as follows: diseases of the digestive system (44.2%) for individuals receiving 5 or fewer treatments, diseases of the skin (60.0%) for those receiving 6 to 10 treatments and diseases of the digestive system (38.4%) for those receiving 11 or more treatments (Table 2).

**Table 2:** Adverse experiences on traditional Korean medicinal therapies and the number of disease incidents

**Demographic characteristics**	**No. of respondents**	**Adverse experience**		**Diseases related to adverse experiences**				
		**No**	**Yes**	**Digestive system**	**Skin**	**Nervous system**	**Kidney**	**Others**
All respondents	915	840(91.8)	75(8.2)	30(40.0)	23(30.6)	11(14.6)	5(6.6)	6(8.0)
Gender								
Male	425	388(91.3)	37(8.7)	16(43.2)	14(37.8)	2(5.4)	3(8.1)	2(5.4)
Female	490	452(92.1)	38(7.9)	14(36.8)	9(23.7)	9(23.7)	2(5.3)	4(10.5)
Age								
20-29	194	174(89.7)	20(10.3)	12(60.0)	5(25.0)	1(5.0)	1(5.0)	1(5.0)
30-39	244	223(91.4)	21(8.6)	6(28.6)	8(38.0)	5(23.8)	1(4.8)	1(4.8)
40-49	229	214(93.4)	15(6.6)	3(20.0)	6(40.1)	2(13.3)	2(13.3)	2(13.3)
50-59	158	143(90.5)	15(9.5)	9(60.2)	1(6.6)	2(13.3)	1(6.6)	2(13.3)
60-69	90	86(95.6)	4(4.4)	-	3(76.0)	1(24.0)	-	-
N. of utilizations								
≤5	675	623(92.3)	52(7.7)	23(44.2)	16(30.8)	6(13.5)	2(3.8)	4(7.7)
6-10	120	110(91.7)	10(8.3)	2(20.0)	6(60.0)	1(10.0)	-	1(10.0)
≥11	120	107(89.2)	13(10.8)	5(38.4)	1(7.7)	3(23.1)	3(23.1)	1(7.7)

All data are in n (%).

## Discussion

This study was conducted to report usage patterns and adverse experiences on TKM targeting consumers in South Korea. We found that of the 2,000 respondents, only 45.8% had used TKM in 2008. Among countries with percentages of TM usage in Northeast Asia in 2001, China, Japan and South Korea had usage percentages of 90.0%, 49.0% and 69.0%, respectively [9]. In contrast, our survey results revealed a decrease in the TM usage rate in South Korea. For individuals over 20 years of age, the usage of TKM was 45.8% compared with the usage by individuals 0-18 years of age at 11.5% [10]. Additionally, the rate of acupuncture use in South Korea was 36.7%, which was much higher than rates in the UK (1.6%), the US (1.1%), and Japan (6.1%) [11]. However, approximately 70% of the individuals used TKM 5 or fewer times.

Among the participants over 20 years of age included in this study, 8.2% reported adverse experiences on TKM. That rate obtained in the present study is higher than the rate of adverse experience of 0.27% reported for CAM in Korean children aged 0 to 18 years [10].

TKM therapies function by stimulating or applying pressure to the skin, muscles, or skeleton of the human body and can cause adverse experiences, such as diseases of the skin and nervous system. The representative adverse experiences of these therapies included pain or itching, burning, bleeding or hematoma and paralysis. Regarding the oral administration of traditional herbal medicines, the digestion, absorption, metabolism and excretion of the medicine can cause adverse events on the digestive and kidney systems, including stomachache, jaundice, liver failure, and oedema of the face, hands and feet. In this survey, acupuncture and herbal medicines were considered the main causes of the adverse experiences observed.

The adverse experiences of acupuncture were primarily diseases of both the skin and nervous system. When assessed using the incidence frequency of the two diseases, the rate of adverse experiences of acupuncture was 3.7%, which is lower than the rates reported in any other country. According to a review of the reported results in databases, the literatures, and randomised clinical trials from countries that are comparable to South Korea, the rates for adverse experiences were 11.8% in Canadian children, 8.6% in Switzerland, and ranged from 6.71% to 15% in China [12-14]. Additionally, when the total incidence rate for adverse experiences among the general population of South Korea was calculated based on the rate of 3.7% found in this study, the rate was 0.04 per 10,000, which is lower than the rate of 0.55 per 10,000 reported in the US [15].

Studies in China have reported that the rate of adverse experiences from acupuncture depends on age and gender, with older age groups and males having higher rates than females [16]. In Japan, younger age groups and females were more sensitive to needle stimulation, while elderly individuals were better able to tolerate pain [11]. In the current study in South Korea, the rate of adverse experiences did not correlate with age; however, males displayed a higher incidence of diseases of the skin, while females had a higher incidence of diseases of the nervous system.

Regarding the relationship between the frequency and adverse experiences of acupuncture, adverse events occurred more often during the early stage of the treatment than during the late stages. We can speculate that this difference occurs because people may discontinue treatment if they experience adverse events during the beginning stages.

In this study, the main adverse experiences due to acupuncture were itchiness, bleeding or hematoma and paralysis; these results are similar to those reported in other studies. The adverse events reported for Canadian children included pain, bruising, bleeding and a worsening of symptoms; Americans experienced sedation (30.98%), needle pain (25.44%), and neuropathy/nervous system-related issues (15.42%); and Swiss patients experienced bleeding or hematoma (6.1%), pain (1.7%) and vegetative symptoms (0.7%) [12,13,17]. However, a Chinese review article found that the adverse events reported in China included symptoms that were more serious, such as pneumothorax, fainting, subarachnoid haemorrhage and infection [14], thereby showing the disparities in the severity of adverse events between countries.

Following acupuncture, the next most common cause of adverse experience was herbal medicine, i.e., Chinese herbs. In Korea, significant adverse effects such as hepatitis and neuropathy were first reported at conferences in 1999 and 2000, respectively [18,19].

The main adverse events were diseases of the digestive system and kidney. The rate of adverse experiences based on the incidence of these two types of diseases was 3.8% or less, and the estimated risk of serious adverse experiences in the overall population was as low as 0.04/10,000 patients in Korea. There were no differences relative to gender, although individuals in their 20s who took herbal medicines more frequently showed diseases of the digestive system as an adverse experience. This is contrary to the case of acupuncture, in which more frequent use resulted in less adverse events. These findings indicate that the adverse experiences of with herbal medicines are more serious. Acupuncture is associated with minor and simple adverse experiences, whereas herbal medicines can be metabolised and cause serious damage to the organs.

In modern society, which requires state-of-the art science in medicine, CAMs have gained popularity. However, tragic adverse events, such as Chinese herbal neuropathy, have already been reported in studies or case reports [20]. In China, there have been frequent reports of adverse events on TM in recent years, but the causes of these events are complex. TMs are thought to damage the liver and kidney by mechanisms similar to those observed for Western medicines [21]. The representative symptoms of herbal medicine adverse experiences found in this study included stomachache, diarrhoea, jaundice, liver malfunction, and oedema of the face, hands and feet.

This study was a survey of the general public and the follow-up of consumers’ adverse experiences was limited; we were unable to determine the root causes and effects of the adverse events. We conducted a survey of the general public regarding TM treatments, the adverse experiences and disease development to assess the types of adverse events. This type of survey method is not as accurate or detailed as research on adverse experiences. However, given the few reported cases of adverse experiences resulting from herbal medicines as well as the absence of reporting systems for other traditional therapy methods, this study was meaningful in that it allowed for the assessment of the current status of the adverse experiences of herbal medicines in the general population of Korea.

## Conclusion

Medical usage and the occurrence of adverse events on TKM should be surveyed periodically and their statistical trends should be analysed. It is necessary to improve the national pharmacovigilance system for spontaneous adverse event reports by reviewing the disparity between the occurrence of herbal medicine reports and the national pharmacovigilance reports in survey data. Policies and regulations are required to enhance the reporting of adverse experiences not only for herbal medicines but also for TKM therapies.

## Competing interests

The authors declare that they have no competing interest.

## Authors’ contributions

HS, SJJ, DSH, BKK and MSL designed the study, performed search, extracted data, carried out analyses and interpretations of the data, and drafted this report. All authors read and approved the final manuscript.

## Pre-publication history

The pre-publication history for this paper can be accessed here:

http://www.biomedcentral.com/1472-6882/13/340/prepub
